# 3D Printed Supercapacitor Exploiting PEDOT-Based Resin and Polymer Gel Electrolyte

**DOI:** 10.3390/polym15122657

**Published:** 2023-06-12

**Authors:** Valentina Bertana, Giorgio Scordo, Elena Camilli, Limeng Ge, Pietro Zaccagnini, Andrea Lamberti, Simone Luigi Marasso, Luciano Scaltrito

**Affiliations:** 1 Department of Applied Science and Technology (DISAT), Polytechnic of Turin, Corso Duca degli Abruzzi 24, 10129 Torino, Italy; elena.camilli@polito.it (E.C.); s263841@studenti.polito.it (L.G.); pietro.zaccagnini@polito.it (P.Z.); andrea.lamberti@polito.it (A.L.); simone.marasso@polito.it (S.L.M.); luciano.scaltrito@polito.it (L.S.); 2 Department of Biotechnology and Biomedicine Nanofabrication, Technical University of Denmark (DTU), Ørsteds Plads, 344, 2800 Kgs. Lyngby, Denmark; 3 Center for Sustainable and Future Technologies, Istituto Italiano di Tecnologia (IIT), Via Livorno 60, 10144 Torino, Italy; 4 CNR IMEM, Parco Area delle Scienze, 37 A, 43124 Parma, Italy

**Keywords:** 3D printing, PEDOT, supercapacitors, energy storage, stereolithography

## Abstract

Renewable energy-based technologies and increasing IoT (Internet of Things) objects population necessarily require proper energy storage devices to exist. In the view of customized and portable devices, Additive Manufacturing (AM) techniques offer the possibility to fabricate 2D to 3D features for functional applications. Among the different AM techniques extensively explored to produce energy storage devices, direct ink writing is one of the most investigated, despite the poor achievable resolution. Herein, we present the development and characterization of an innovative resin which can be employed in a micrometric precision stereolithography (SL) 3D printing process for the fabrication of a supercapacitor (SC). Poly(3,4-ethylenedioxythiophene) (PEDOT), a conductive polymer, was mixed with poly(ethylene glycol) diacrylate (PEGDA), to get a printable and UV curable conductive composite material. The 3D printed electrodes were electrically and electrochemically investigated in an interdigitated device architecture. The electrical conductivity of the resin falls within the range of conductive polymers with 200 mS/cm and the 0.68 µWh/cm^2^ printed device energy density falls within the literature range.

## 1. Introduction

Nowadays, the new generation of energy storage systems has more demanding requirements in terms of compactness, portability, integration, especially for integrated electronics requiring the development of miniaturized devices, energy and power densities, and environmental compliance [1,2,3,4]. One of the most promising energy storage system that represent a complementary technology to the better-known rechargeable batteries is the micro electrochemical supercapacitor (MSC). MSCs are very interesting candidates for storing energy thanks to their versatile voltage window and power densities, making them applicable in different scenarios as energy buffers. In the field of MSCs, three main categories of devices exist, namely electrochemical double-layer capacitors (EDLCs) [5], pseudocapacitors (PCs) [6] and battery-hybrid technologies [7,8]. In the last decades, several groups are aiming to miniaturize the energy storage devices that are developed through standard fabrication techniques such as the slurry-coating [9], or spray-coating methods [10]. However, with the standard approach, several complex processing steps [11,12] are required which makes the integration of MSCs in portable devices (like wearable electronics, and smart medical sensors) extremely challenging [13]. Against this backdrop, additive manufacturing (such as 3D printing) is a promising technology for the fabrication of integrated devices allowing the selective deposition of customized materials in potentially any shape. Moreover, moving from planar to three-dimensional geometry gives the incredible possibility to increase the energy storage devices electrodes active area, also allowing the production of complex geometries and flexible devices [14]. Three common strategies have been reported in the recent literature to fabricate a 3D SC device [12,15,16]: filament deposition (FDM) [17], direct ink writing (DIW) [18,19] and inkjet printing (InkJet) [20,21]. Despite the advantages previously mentioned, FDM and DIW show many issues in providing high-resolution and large-volume objects at the same time, making them less suitable for commercial applications. For this reason, over the last decade, stereolithography (SL) or digital light processing (DLP) have become widely used for 3D energy storage device production [22,23,24]. These technologies allow the best performances in terms of complexity, resolution, and shape accuracy. Moreover, new designs and improved performances are achieved with new classes of photocurable composite resins based on nanomaterials [25,26,27] outlining a promising approach for portable flexible electronics. Moreover, the huge number of printable materials and printing techniques available on the market provide the possibility to manufacture complex 3D structures with attractive electrical [24], chemical [28], and morphological [20,29] properties. As regards the materials, a large and growing body of literature has investigated their role in SCs fabrication [30]. In this regard, carbon materials are one of the most intensively investigated [31,32], due to their high electrical conductivity, good electrochemical stability, and low cost. Conductive polymers (CP), such as Polythiophenes (Pth) [33], Polyaniline (PANI) [34] and Polypyrrole (PPy) [35], are also widely adopted due to their good conductivity, ease of synthesis, and high intrinsic flexibility and adhesion on different substrates [36]. For example the Poly(3,4-ethylene dioxythiophene (PEDOT) and PANI [37,38] fillers are the most used to produce 3D SCs devices. On the other hand, the main non-polymeric fillers for SL or DLP resins used for printing 3D SCs are: carbon-based materials (e.g., reduced graphene oxide (rGO) [27,38] or multiwall carbon nanotube (MWCNT) [39]) and metal powders (e.g., silver nanoparticles (AgNPs) [40]). Although many printable conductive resin formulations are reported in literature, they are not so close to the ideal performances required for an efficient electrochemical capacitor like high electrical conductivity, chemical stability, and extensive longevity after the charge-discharge cycles [37,38,41]. In recent years, there has been an increasing amount of literature on the PEDOT polymer application in energy storage devices due to its high ionic and electronic conductivity and good thermal stability [42,43]. One advantage of PEDOT is the possibility to deposit it as a continuous conductive layer without any need for a metallic substrate, as reported in recent work by Wang et. al. [44]. An issue of CPs, when exploited in energy storage devices, is their low mechanical stability due to the volume change happening during the charging and discharging phases. This required the CPs to be inserted into structural scaffolds like natural celluloses [45,46,47,48].

Our work aims to introduce an innovative and integrated SCs device composed of a 3D printable electrically conductive polymeric blend [49] based on Poly(3,4-ethylenedioxythiophene)–poly(styrene sulfonate) (PEDOT:PSS), exploiting a ceramic substrate. Herein, the SCs electrodes were obtained using a custom SL printer able to print features down to 500 μm [50] and less [51], as also reported in the Supplementary Information (see Figures S4 and S5). The behaviour of the 3D printed SCs based on PEDOT:PSS was characterized by cyclic voltammetry and galvanostatic charge-discharge measurements. Among the proposed designs, one allowing to reach an areal capacitance of 11.4 mF/cm^2^ was identified, perfectly in line if not better than other devices found in the literature. Furthermore, the long-term charge/discharge characteristics were investigated to evaluate the stability of the proposed 3D energy storage device. It turned out that the proposed device is fully compliant for ultra-low power energy harvesting and storage application. Therefore, the printable PEGDA-based resin represents a solution for printing integrated energy storage devices in more complex 3D printed systems. Since the purpose of the present study was the characterization of the PEDOT-based resin as a supercapacitor (which is novel in this field, as reported in [52]), the geometrical requirements were relaxed allowing an easier fabrication process and a more stable device assembly. A Polydimethylsiloxane (PDMS) packaging was then added to protect and prevent the evaporation of the electrolyte gel employed during the characterizations.

## 2. Materials and Methods

Poly(ethylene glycol) diacrylate (PEGDA 250) (average molecular weight Mn 250), was supplied from Sigma Aldrich (Saint Louis, MO, USA); a commercial solution of PEDOT:PSS Poly(3,4-ethylenedioxythiophene)–poly(styrene sulfonate) was purchased from Heraeus (Hanau, Germany; product name: Clevios^TM^ PH 1000). The radical photoinitiator powder bis(2,4,6-trimethylbenzoyl)-phenylphosphineoxide (Irgacure 819) was provided by BASF Schweiz AG (Kaisten, Switzerland). Poly(vinyl alcohol) (PVA, Mw ≈ 95000 g/mol), and KCl (85 wt. % aqueous solution) were obtained from Sigma Aldrich. Polydimethylsiloxane (PDMS) was purchased as a two-component kit containing the vinyl-terminated base (Sylgard 184) and curing agent both from Gelest Inc. (Morrisville, PA, USA).

### 2.1. PEGDA:PEDOT Resin Preparation

The PEDOT:PSS filler was prepared from a commercial solution, Clevios™ PH1000, slightly modifying the procedure previously reported by Scordo et al. [49]. Clevios™ PH1000 was dispersed in a 0.5 M H_2_SO_4_ solution in a 1:10 ratio. After 24 h, the PEDOT:PSS precipitate was extracted by centrifugation, and the leftover was diluted in double weight of ethanol, whose low density facilitates the particle suspension [51]. In order to enhance the separation of the aggregates, the solution was treated with a digital sonifier (Branson Ultrasonics Sonifier SFX250 Cell Disruptors) at 65% power for 10 min (5 s ON and 5 s OFF) and thereafter with Ultra-Turrax^®^ (IKA, model T25, Staufen, Germany) homogenizer for 20 min at 30,000 rpm. After overnight incubation, the supernatant was separated and discarded by two consecutive centrifugations, at 4000 rpm for 20 min, and at 5000 rpm for 20 min. The sample accordingly produced is named treated PEDOT:PSS and appears as a compact, blue, wet powder.

The resin matrix was then produced by adding 1% wt. of Irgacure 819 in PEGDA 250; the photoinitiator dispersion was enhanced using the Branson sonifier at 30% power for 10 min (5 s ON and 5 s OFF) in an ice bath. Finally, the photocurable conductive blend was obtained by mixing the treated PEDOT:PSS with the resin matrix, using the Branson sonifier at 30% power for 10 min (5 s ON and 5 s OFF) in an ice bath. From now on, the so prepared PEDOT-based resin is also called PEGDA:PEDOT resin.

### 2.2. Device Design

The SCs object of the present work was firstly designed in Computer-Aided Design based software (SolidWorks) (See Figure S1). It consists of two three-dimensional interdigitated electrodes (3D IDE), as reported elsewhere in the literature [38,53,54]. It was grown on a 21 × 12 mm^2^ area and is made up of 1 mm wide fingers, 1 mm apart from each other. Three electrode thicknesses were considered: 0.5 mm, 1 mm, 2 mm.

A metallic collector was designed to enhance the electrode’s contact. Such a collector was obtained by platinum (Pt) sputtering through a hard mask having the same geometry of the 3D IDE. Then, a PDMS chamber was designed to avoid electrolyte evaporation during long-term measurements.

### 2.3. D Printing and Device Assembly

A custom SL printer (Microla Optoelectronics s.r.l., Turin, Italy) was used to obtain the 3D printed SCs (See Figure S3 in Supplementary Information for more details). The printer mounts a 405 nm wavelength continuous laser and two dedicated boards for laser control and motion control. Movements inside the printer include the immersion of the platform inside the resin vat and the doctor blade swiping on the platform. The layer thickness was set to 100 μm, the laser scan velocity and nominal output power were 1000 mm/s and 40 mW respectively. After the printing process, the 3D device was rinsed by 2-Propanol alcohol to remove unreacted resin residues. Then, a curing process was conducted under UV lamp (Hamamatsu Spot light source LC8) for 5 min at 100% power (10 mW/cm^2^) in inert atmosphere. Finally, the 3D IDE electrodes were annealed and at the same time assembled on the substrate with the chamber. The annealing consists in heating up the SCs assembly at 120 °C in vacuum oven, letting them for 1 h, and finally cooling down to ambient temperature. This process demonstrated to be beneficial for PEGDA:PEDOT resin performance, as reported in [51].

As beforementioned, the 3D IDE was connected to a Pt collector. An alumina flat substrate was chosen as inert material to sputter the collector and then fix the 3D IDE. Such an alumina flat substrate was previously etched following the shape of the IDE to improve their assembly and ensure finger distance during tests. The alumina etching process was performed with a 50 W infrared pulsed laser obtaining a 200 μm etching depth (laser process by Microla Optoelectronics s.r.l, Turin, Italy). Platinum was then sputtered on the ceramic substrate by Q150T S sputter coater from Quorum Technologies with Argon plasma. A thin layer of resin was deposited on a glass slide by spinning the PEGDA:PEDOT resin at 1000 rpm for 60 s. Then, the 3D-printed electrodes were softly pressed on the film resin to cover their bottom side with the conductive blend. The thin resulting layer of resin will penetrate the Pt-coated alumina substrate pores and the porous PEGDA:PEDOT printed electrodes, enhancing the electrode-collector contact. Then, the 3D IDEs were aligned on the platinum-sputtered substrate under a microscope and immediately cured under the same UV light used for post-curing for a few seconds. The complete bonding occurred during the subsequent annealing step.

### 2.4. Electrochemical Measurements

Electrochemical measurements were carried out by means of VMP 3, provided by BioLogic, for cyclic voltammetry (CV), galvanostatic charge and discharge (GCD), and electrochemical impedance spectroscopy (EIS) measurements, while the BT2000 cycler, provided by Arbin, was mainly exploited for cycling stability measurements.

CVs were run to evaluate the current-voltage characteristic of the device, as proof of the capacitive behaviour. The scan rates were {1, 2, 5, 10} mV/s, and the voltage window was set to 0.8 V. The voltage resolution was 100 µV but the current was recorded 100% over 10 averaged voltage steps, hence the actual step amplitude was 1 mV.

In the same voltage window, GCD tests were run at the current rates of {5, 10, 20, 50, 100} µA/cm^2^ to evaluate the rate capabilities and to extract the energetic characteristics to be reported in the Ragone plot. Here the sampling conditions were 5 mV of voltage variation.

Electrical quantities were evaluated as follows. Energy and charge were evaluated directly from the measured quantities:

$$E=\int_{\Delta t}^{}i\left(t\right)v\left(t\right)\;\mathrm{dt}$$

$$Q=\int_{\Delta t}^{}i\left(t\right)\;\mathrm{dt}$$

where v(t) and i(t) are the voltage and current signals, respectively. The evaluations were carried out in the semi-periods Δt of charge and discharge, then charging and discharging quantities were used to evaluate the respective efficiencies:

$$\eta_{C}=\frac{Q_{d}}{Q_{c}}$$

$$\eta_{E}=\frac{E_{d}}{E_{c}}$$

Capacitance was evaluated through energy and charge according to

$$C_{d}=\frac{1}{2}\frac{Q^{2}}{E}$$

Finally, the average discharge power was calculated according to:

(1) $$P=\frac{E_{d}}{\Delta t_{d}}$$

EIS measurements were performed by applying a 5 mV amplitude perturbation of varying frequency in the range [5 × 10^5^, 1 × 10^−2^] Hz. The frequency range was sampled ten points per decade.

## 3. Results

### 3.1. PEGDA:PEDOT Resin Characterization

**Figure 1:**
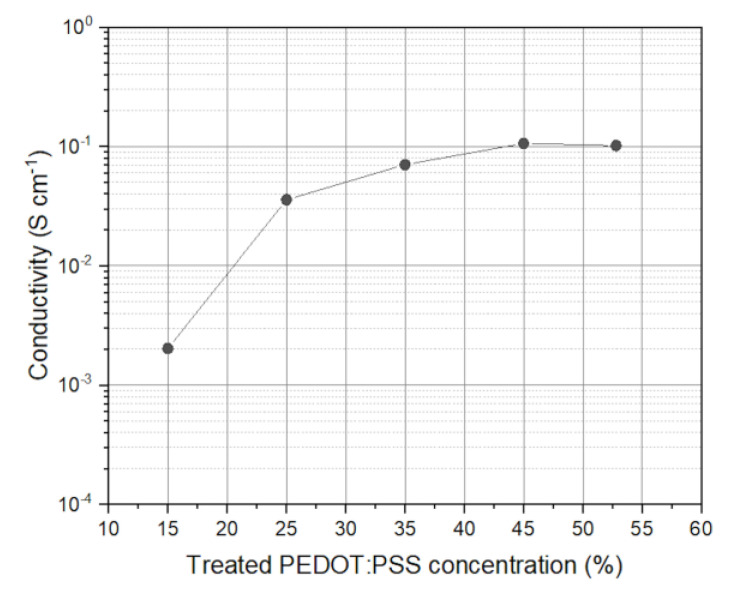
Conductivity profile with respect to treated PEDOT:PSS wt.% added in the PEGDA 250 polymer for PEGDA:PEDOT resin preparation.

The PEGDA:PEDOT resin was examined so to get the best compromise between printability and electrical conductivity. Electrical conductivity vs. treated PEDOT:PSS concentration is reported in Figure 1. Such characterization was performed to confirm the previously found results [55]. Therefore, single samples (0.5 × 0.5 × 1 cm^3^ cuboid) were prepared for different treated PEDOT:PSS percentages in PEGDA:PEDOT resin. The material conductivity was then calculated from resistance measurements. In the explored percentage amounts, the conductivity ranged within 1 mS/cm at low percentages up to 100 mS/cm at 50% treated PEDOT:PSS content. This is perfectly in line with the previously obtained results [55]. Above these levels, the composite material couldn’t be printed through stereolithography, due to the excessive viscosity of the PEGDA:PEDOT resin which does not allow a proper resin recoating in the considered printing setup. The obtained conductivity is remarkable considering that the electrical conductivity is about 200 mS/cm for PEDOT:PSS [56] while for good PEDOT films, it can reach up to 5400 S/cm [57]. This represents an advantage in the choice of the conductive polymer, considering that other conductive polymers show lower conductivities, as in the case of polypirrole with 10^−3^ S/cm [58].

Other characterizations of PEGDA:PEDOT resin are reported in [49,51,55,59].

**Figure 2:**
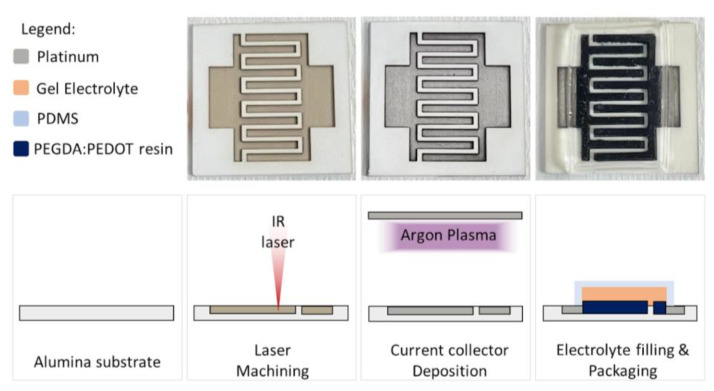
Device assembly procedure. From the top, top view of real device and schematic cross-sectional views.

As regards the final device, Figure 2 reports the picture of it at each fabrication step, from alumina laser machining to final packaging. In this process the PEGDA:PEDOT resin was also used as adhesive layer between the 3D printed device and the ceramic rigid substrate providing a good mechanical stability and conductivity. Figure S2 shows the device during testing.

### 3.2. Device Performance

**Figure 3:**
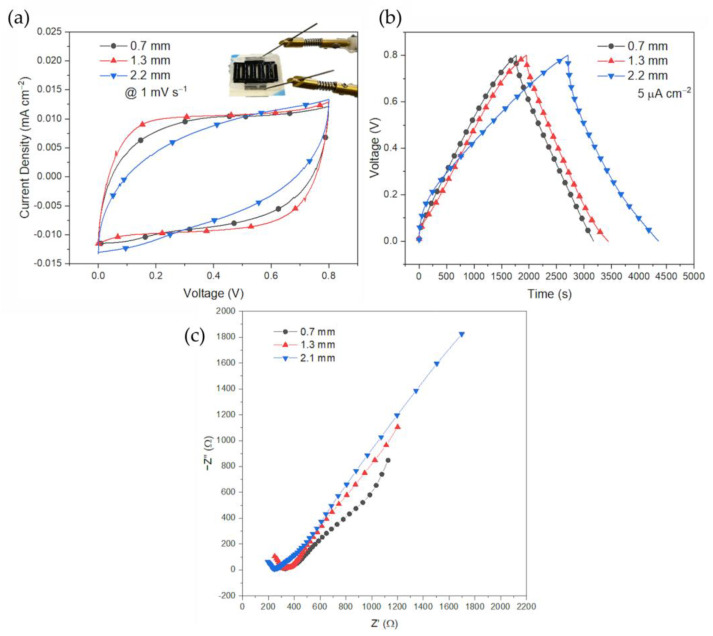
Cyclic voltammetry in (**a**) and galvanostatic profiles in (**b**) with respect to the different printed electrodes thicknesses 0.7 mm, 1.3 mm, and 2.1 mm. CVs are reported for the scan rate of 1 mV s^−1^ and GCDs for the current rate of 5 µA cm^−2^. In (**c**) the EIS spectrums for the three different thicknesses show an increasing trend in the diffusion length with the increasing electrode thicknesses.

Electrodes with different thicknesses were printed and tested to evaluate the scaling properties of the PEGDA:PEDOT resin. After printing, the actual IDE thicknesses were 0.7 mm, 1.3 mm and 2.1 mm (more details about these results can be found in the Supplementary Information, see Figure S6). Symmetrical devices were tested by means of potentiostatic and galvanostatic techniques at different rates. The results of the lowest rates are reported in Figure 3. In Figure 3a it is possible to observe the CV carried out at 1 mV/s. The CV shape is capacitive in all cases, although resistive as suggested by the smoothed edges. Capacitances are close, especially for the low thicknesses. The behaviour at the maximum electrode thickness is more resistive as expected by the conductivity of the resin. In Figure 3b the voltage profiles of the low 5 µA/cm^2^ GCD test are shown. The rated capacitances were 10.5 mF/cm^2^, 11.4 mF/cm^2^, and 19.5 mF/cm^2^ for the increasing thicknesses. With these results, it is possible to confirm that for the 0.7 mm and 1.3 mm cases, capacitances are close since the discharge slopes are comparable. Hence, it can be deduced that these thicknesses represent a limiting value for the maximum areal capacitance that can be get from this material with acceptable yield and power capabilities. Above these limits, the material increases its losses as suggested by the galvanostatic profile of the device assembled with the 2.1 mm thick electrodes. Here the 2.1 mm device shows a not efficient working point as suggested by the relatively low coulombic efficiency. Since the system is working within the voltage limit of the electrolyte, the charging period can be altered by the presence of leakage currents due to slow and intense redistribution phenomena over the whole electrode’s length. At the fixed current rate used for the comparison, these increased leakages strongly oppose the charging phase leading to a prolonged charging time.

Device resistances were investigated via EIS as reported in Figure 3c. In this plot, it is evident how increasing the electrode thickness increases the diffusion length akin to a slower device [60]. Further, the result confirms that the device capacitances are similar in the low-thickness cases as suggested by the low values of the imaginary parts at the limiting low frequency. The high value of the reactance of the 2.1 mm thick device suggests that other than being more resistive, the material is not fully exploited for energy storage, meaning that thicker electrodes are not convenient.

**Figure 4:**
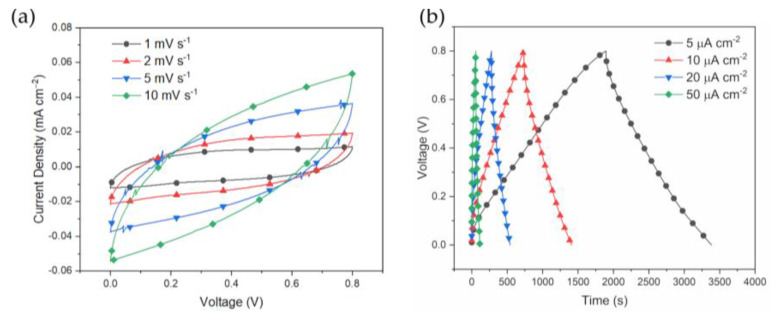
Cyclic voltammetry in (**a**) and galvanostatic profiles in (**b**) at fixed printed electrodes thickness of 0.7 mm. CVs are reported for the scan rates of {1, 2, 5, 10} mV s^−1^ and GCDs for the current rates of {5, 10, 20, 50} µA cm^−2^.

The rate capabilities were examined for the 3D-printed devices. Results are reported in Figure 4a,b in which rate capabilities were studied either in potentiostatic or galvanostatic modes, respectively. The resistive behavior of the electrodes is more evident by the CV results of Figure 4a. Here, for scan rates above 2 mV/s the device behaves resistively, hence with reduced storage capabilities. This is evident from Figure 4b since the IR-drop increases from 10 mV at 5 µA/cm^2^ to 100 mV at the 10 times higher current rate, as expected.

**Figure 5:**
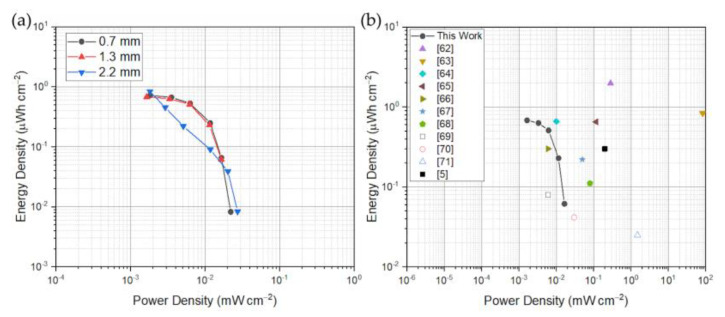
(**a**) the Ragone plot evaluated for the devices printed with different electrodes thicknesses of 0.7 mm, 1.3 mm and 2.1 mm, in (**b**) a comparison between the 1.3 mm thick device with literature reports.

The Ragone plot of Figure 5a was evaluated out of the GCD rate capability tests. According to the map proposed in [61], the here proposed device is fully compliant for ultra-low power energy harvesting and storage application. As previously claimed, the thicker electrode device shows its dissipative effects as the stored energy is strongly reduced at fixed charging power. Hence, this solution shall be avoided. Stored energy densities were on the order of 0.7 µWh/cm^2^ at low power density values of 1 µW/cm^2^ while they were dropping to low energy density values of 60 nWh/cm^2^ c.a. at power density levels of 20 µW/cm^2^. In Figure 5b the rated performances are represented with literature results. Although the low power capabilities of the produced SC, the energy density falls in the range of other additive manufactured devices [5,62,63,64,65,66,67,68,69,70,71]. The values considered for comparison in the plot of Figure 5b are reported in Table S1. The comparison was done with devices obtained with different fabrication processes and different active materials compositions.

**Figure 6:**
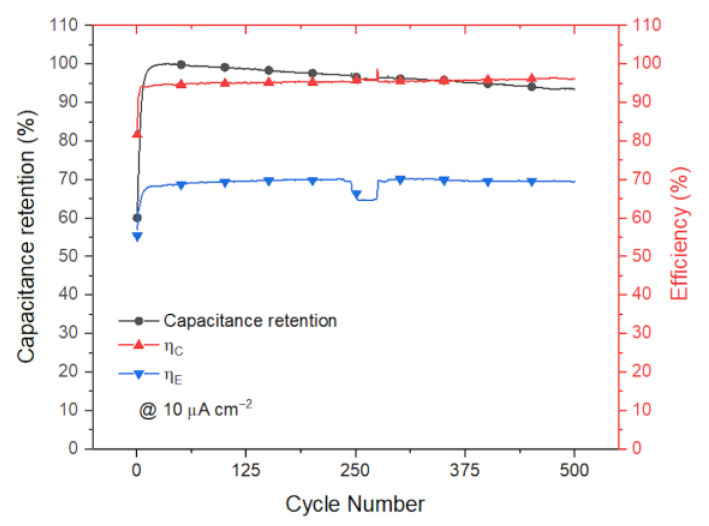
Cycling stability carried out by cycling the device at the low rate of 10 µA cm^−2^. Capacitance retention, left axis, and efficiencies, right axis, are reported for the voltage window of 0.8 V.

Cycling stability was evaluated via galvanostatic cycling at a relatively low current rate of 10 µA/cm^2^. At this current rate, the cycle period takes around 1500 s, as can be appreciated from the plot of Figure 6. In this plot, capacitance retention and coulombic and energetic efficiencies are reported versus the number of cycles. The measurement lasted more than 10 days, and the device retained more than 90% of its initial capacitance. Coulombic and energetic efficiencies were increasing over time: coulombic efficiency reached more than 95% while energetic efficiency approached almost 70%. After 500 cycles, the electrolyte started drying faster and we reconducted this to failures in the PDMS packaging. We addressed the sloping in the slow drying of the electrolyte due to the PDMS permeability.

## 4. Conclusions

In this work, a novel composite material was proposed for stereolithographic printing method to print conductive geometries. The photocurable composite material was composed of treated poly(3,4-ethylenedioxythiophene): polystyrene sulfonate (PEDOT:PSS) blended with poly(ethylene glycol)diacrylate (PEGDA) resin. The composition was optimized to get a good compromise between printability and electrical conductivity of the printed geometries. Electrodes for energy storage application were printed to fabricate interdigitated supercapacitors, whose electrolyte was jellified aqueous KCl in a PVA matrix. Different electrode thicknesses were 3D printed to study the scaling properties of the proposed novel material. It was observed that due to the relatively high 3D printed PEGDA:PEDOT resin resistivity, electrode thicknesses above 1.5 mm suffer from ohmic losses, hence, low aspect ratio devices can be printed to get devices with energy and power densities of 0.7 µWh/cm^2^ and 20 µW/cm^2^. These results are in line in terms of energy density with the literature performance of 3D printed devices obtained with different fabrication methods.

Even if the 3D printed supercapacitor object of this paper has room for improvement, especially as regards the power capabilities, it represents a step forward in the fabrication of supercapacitors, since a new and potentially biocompatible material was employed. One of researchers’ wish in the field of wearable electronics or IoT compatible devices is to reach a high customization degree with a flexible fabrication process. Such request can be fulfilled by using 3D printing in combination with the material object of this work. Indeed, in case the demand of current and power is low, the PEGDA:PEDOT resin would allow to print both the conductive path and the energy storage component, if the circuit is properly designed. Not least, applications in 3D printed Micro Electro Mechanical Systems (MEMs) are promising.

## Data Availability

Data is contained within the article or supplementary material.

## Supplementary Materials

The following supporting information can be downloaded at: https://www.mdpi.com/article/10.3390/polym15122657/s1. Figure S1. CAD image of the PEGDA:PEDOT 3D printed SC components: (a) the electrodes (dimensions are expressed in mm); (b) the collector (yellow) and the PDMS chamber (grey). Figure S2. C device during testing. Figure S3. (a) Microla stereolithography printer, (b) printing platform, (c) adjustable doctor blade. Figure S4. Pattern test geometry: (a) features diameters; (b) CAD of the pattern test geometry; (c) features heights. Figure S5. Pattern test geometry printed with Standard Blend resin in Microla SL printer. Figure S6. The three IDE thicknesses: 0.7 mm, 1.3 mm, 2.2 mm. The bending is due to polymer shrinkage after curing. The bending effect is compensated when the electrodes are fixed to the alumina substrate. Table S1 Performances of supercapacitors from literature
