# Supplementary material for: 3D Printed Supercapacitor Exploiting PEDOT-Based Resin and Polymer Gel Electrolyte
Source: Polymers (Basel). 2023 Jun 12;15(12):2657. doi: 10.3390/polym15122657 (PMC10305262; doi:10.3390/polym15122657)
Supplement: Supplementary file 1 [file polymers-15-02657-s001.zip › polymers-2407332-supplementary.pdf]

polymers

MDPI

Supplementary Material: 3D Printed Supercapacitor Exploiting PEDOT-Based Resin and Polymer Gel Electrolyte

V. Bertana, G. Scordo, E. Camilli, L. Ge, P. Zaccagnini, A. Lamberti, S. L. Marasso and L. Scaltrito

Electrodes design

As stated in the main paper, the design of the supercapacitor is based on an interdigitated geometry with 1 mm wide fingers. Electrodes printing and hard mask for sputtering the current collector follow this same geometry. Sputtering occurs on an insulating alumina substrate, on which the electrodes are fixed. The sputtered collector follows the same IDE geometry and includes a 2 mm additional area to allow the probes connection during testing (Figure 2S). An enclosing chamber (1 mm thick and 2 mm high) is added to hold in place the gel electrolyte. A rectangular cover is used to seal and avoid the electrolyte evaporation. As it is shown in Figure 1S, the two electrodes are blue, the chamber is light grey, and the collector is dark yellow.

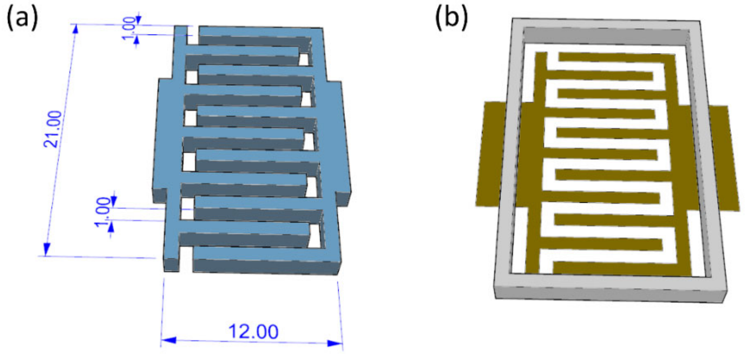

Figure S1. CAD image of the PEGDA:PEDOT 3D printed SC components: (**a**) the electrodes (dimensions are expressed in mm); (**b**) the collector (yellow) and the PDMS chamber (grey).

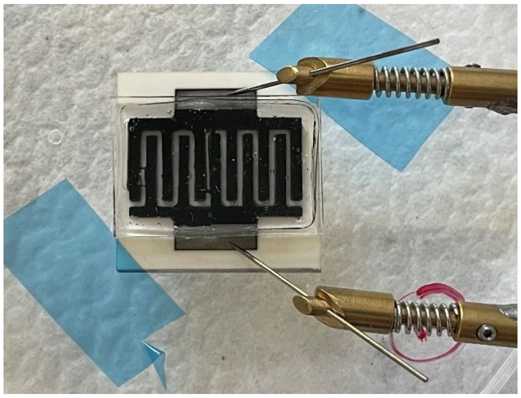

Figure S2. C device during testing.

Electrodes 3D printing

A customized stereolithography 3D printer (Microla Optoelectronics Srl) was employed. It is a 405nm wavelength laser scanning stereolithography printer, with a maximum printing area of 170 x 200 mm^2. The laser spot movement on the printing platform is managed by a galvanometric mirror scanner. A motorized stage controls the upwards and downwards movement of the printing platform at the right polymerization position. After each layer is printed, the platform is dipped in the resin vat; once the fresh materials flows on it, a doctor blade uniforms the resin layer and removes the surplus resin. The doctor blade position, as well as the initial printing platform position, are adjustable in this printer to allow the fabrication of objects also on existing substrates. Figure 3S shows the employed printing setup.

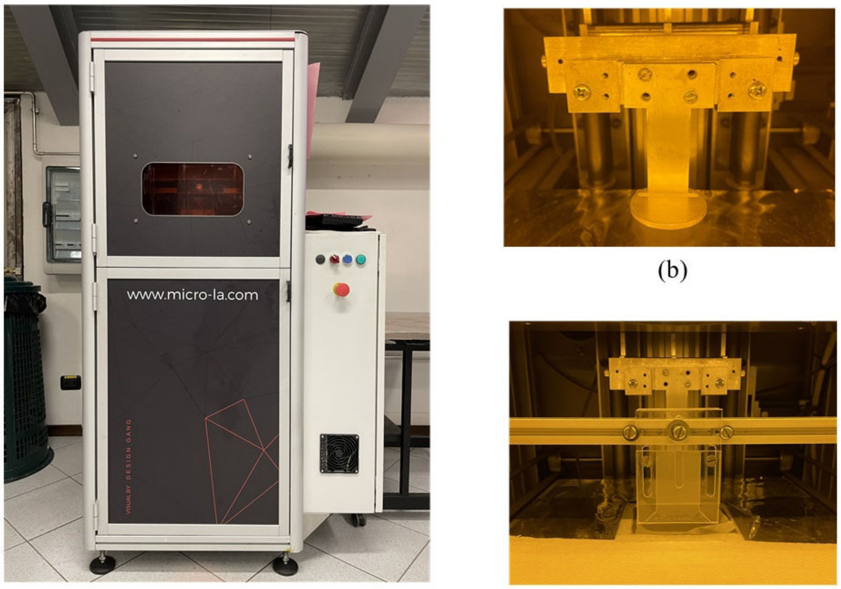

(a) (b) (c)

Figure S3. (**a**) Microla stereolithography printer, (**b**) printing platform, (**c**) adjustable doctor blade.

Pattern test for determining the accuracy of Microla 3D printer

A pattern test is performed to establish the accuracy of the Microla 3D printer. It consists in the fabrication of a geometry with variable features with a commercial resin, namely the FunToDo Standard Blend. The geometry consists of a base on which 12 cylinders and 3 hemispheres are placed. The cylinders have different diameters, ranging from 0.2 to 3 mm, and are 1 mm and 3 mm high. The hemispheres have different diameters, ranging from 1 to 3 mm, and heights from 0.5 to 1.5 mm (see Figure 4S). The Standard Blend commercial resin was chosen since it is dark shaded and resembles the PEGDA:PEDOT resin.

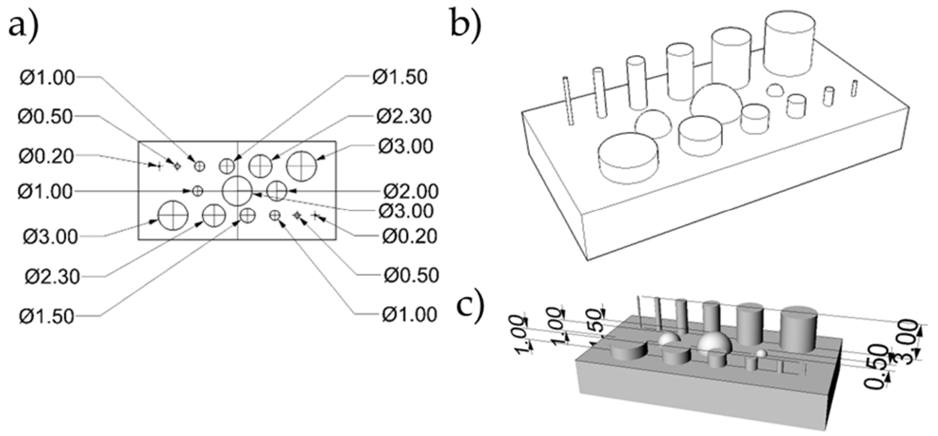

Figure S4. Pattern test geometry: (**a**) features diameters; (**b**) CAD of the pattern test geometry; c) features heights.

As shown in Figure 5S, the geometry was correctly printed, including the tiniest features (200 µm diameter pillars), which were printed with a deviation from the nominal value of 5%.

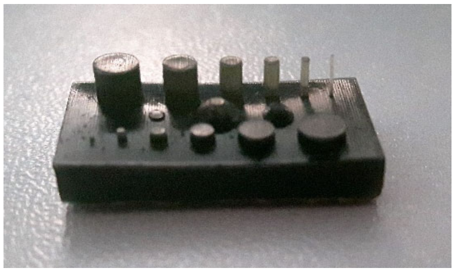

Figure S5. Pattern test geometry printed with Standard Blend resin in Microla SL printer.

Moreover, this printer was demonstrated to be valid for the printing of tiny complex objects, like a 500 µm diameter coil, as reported in [50]Fare clic o toccare qui per immettere il testo..

Electrodes printing accuracy

The printer employed for electrodes fabrication is characterized by a laser spot of almost 80 µm. This means that micrometric features are printable, especially on the XY plane (orthogonal to laser direction) where resolution is maximum. However, as often happens with light-induced polymerization, the resolution along Z axis (parallel to laser direction) is worse, due to the typical ovoid polymerized voxel. Nonetheless, when dealing with part thicknesses near the layer thickness value, the dimensional error on final part can be relevant. Finally, the presence of inclusions like treated PEDOT particles, can furtherly be detrimental to the final obtained part thickness. For these reasons, the final printed IDEs thicknesses are 0.7 mm, 1.3 mm and 2.1 mm with a deviation from nominal values of 0.5 mm, 1 mm, 2 mm. This is perfectly in line with the expectations: the more the IDE thickness is far from the layer thickness, the less the error is (being respectively 40%, 30% and 5% far from nominal value).

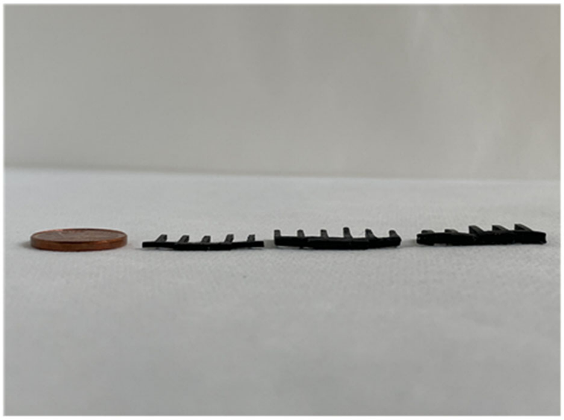

Figure S6. The three IDE thicknesses: 0.7 mm, 1.3 mm, 2.2 mm. The bending is due to polymer shrinkage after curing. The bending effect is compensated when the electrodes are fixed to the alumina substrate.

Performances of supercapacitors from literature

Table S1. Performances of supercapacitors from literature.

Process	Electrodes Materials	Electrolyte	Voltage [V]	Areal Capacitance [mF/cm²]	Energy Density [µWh/cm²]	Power Density [mW/cm²]	Ref.
Selective Laser Melting	PPy on Ti	PVA-H_3PO_4	0.5	57	1.98	0.285	[62]
Cleanroom process	CDC	1M Et4NBF4 in PC	2.0	1.5	0.83	84	[63]
StereoLithography	PEGDA: PEDOT resin	PVA-KCl	0.8	11.4	0.68	0.002	This Work
Sputter/Spray coating	MnO_2-rGO	PVA-H_3PO_4	0.8	7.43	0.66	0.01	[64]
Ink extrusion via micronozzle	CNT	PVA-H_3PO_4	1.0	4.7	0.65	0.117	[65]
Laser	LIG + AC	1M KCl	0.8	25	0.3	0.2	[5]
Spray coating	MWCNT	PEGDA/ [EMIM] [TFSI]	2.2	0.5	0.3	0.006	[66]
Cleanroom process	SiC	1M KCl	0.8	1.7	0.22	0.05	[67]
Electrospinning	CNT + PVDF	PVA-H_3PO_4	0.5	3.2	0.111	0.08	[68]
Electrodeposition	Graphene QD	0.5M Na_2SO_4	1.0	0.47	0.08	0.006	[69]
Digital Light Processing	AgNW	PVA-$LiClO_4$	1.0	0.301	0.042	0.03	[70]
Cleanroom process	N poly-Si	1M H_2SO_4	0.7	0.74	0.025	1.50	[71]
